# Thermal and Electromagnetic Properties of Polymer Holey Structures Produced by Additive Manufacturing

**DOI:** 10.3390/polym12122892

**Published:** 2020-12-02

**Authors:** Philippe Lambin, Aliaksandr Liubimau, Dzmitry Bychanok, Luca Vitale, Polina Kuzhir

**Affiliations:** 1 Department of Physics, University of Namur, B-5000 Namur, Belgium; 2 Higher Education Pedagogical Institute, Bukavu, Congo; 3 Institute for Nuclear Problems, Belarusian State University, 220030 Minsk, Belarus; lubimov@belstu.by (A.L.); dzmitrybychanok@yandex.by (D.B.); polina.kuzhir@uef.fi (P.K.); 4 Radioelectronics Department, Faculty of Radiophysics, Tomsk State University, 634050 Tomsk, Russia; 5 Narrando srl and Department of Industrial Engineering, University of Salerno, I-84084 Fisciano, Italy; luca94univ@gmail.com; 6 Institute of Photonics, University of Eastern Finland, FI-80100 Joensuu, Finland

**Keywords:** nanocomposite, thermal conductivity, microwave shielding, truss lattice, foam

## Abstract

Multifunctional 3D-printed holey structures made of composite polymers loaded with nanocarbon were designed to serve simultaneously as GHz-radiation absorbing layers and heat conductors. The geometry of the structures was devised to allow heat to be easily transferred through, with special attention paid to thermal conductivity. Numerical calculations and a simple homogenization theory were conducted in parallel to address this property. Different structures have been considered and compared. The electromagnetic shielding effectiveness of the produced holey structures was measured in the microwave range.

## 1. Introduction

Holey-structured materials have applications in such various fields as mechanical engineering [1], ultrasonic imaging [2], acoustical [3] and microwave [4] stop-band layers, etc. In addition to intrinsic properties governed by their structures, these materials are lightweight and permeable to both air and visible light. Polymer holey structures offer the additional advantage of being low cost and easy to fabricate. If the polymer is a composite that conducts electricity, it can be used to protect sensitive devices against electromagnetic perturbations [5], which has become a crucial issue in the domain of electromagnetic compatibility [6]. For this kind of application, one is seeking thin and light layers that absorb electromagnetic radiations rather than reflecting them back [7]. It is known that specially designed structures made of polymer containing conducting nanocarbons may absorb microwave radiations efficiently while behaving as poor reflectors [8]. By contrast, metallic structures are less interesting as soon as they reflect more than they absorb. Thus, large electrical DC conductivity is not a necessary condition for high absorbance [9]. Commercial applications of a shielding layer requires an attenuation of the incident electromagnetic power better than 20 db [10].

When a high-power electronic device is shielded against microwave perturbations, it is important that the heat it produces be evacuated through the protecting layer. This is where holey structures offer the advantage of being permeable to air. Thermal regulation of the device will be easier to achieve if in addition to convection, the structure conducts heat well. One is therefore seeking good electrical and thermal conductivity of the polymer [11,12]. Another constraint put on the material is that it should be suitable for 3D-printing, especially via the fused deposition modeling (FDM) technology [12,13,14,15]. The principal reason for that is to be able to build prototypes of the most promising structures and use these models for experimental measurements. The choice was therefore set on thermoplastic polymers, such as polylactic acid (PLA), linear low-density polyethylene (LLDPE), acrylonitrile butadiene styrene (ABS), and composites of them [16].

Low carbon footprint and recycling or reusing plastic wastes has become an issue of paramount importance due to the widespread use of polymers, including in additive manufacturing [17]. Precisely, the polymers just cited offer several nature-friendly characteristics. In particular, PLA is a bioplastic that has fewer environmental effects than petroleum-based polymers. Low-density polyethylene is a very common plastic that can be recycled by different techniques [18]. PLA and ABS, the two most popular polymers used in FDM technology, can be recycled and reused as filaments for a new cycle of 3D printing, without significant degradation of mechanical behavior [19,20]. The cited studies indicate that 3D manufacturing might rely on recycled polymers as source materials, therefore consuming some plastic wastes, failed 3D prints, and also 3D-printed parts at their end of use.

**Figure 1:**
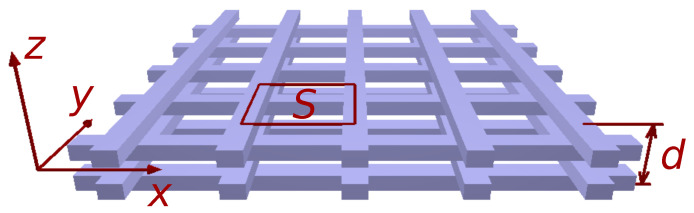
Example of a 2D-periodic holey slab, here composed of two layers of the so-called woodpile structure. This model idealizes structures easily produced by 3D-printing [24]. The unit cell projected onto the horizontal (x,y) plane is the surface *S*. The thickness of the slab is *d*, here twice the height of the unit cell.

The main topic of the present paper is to compute the effective thermal conductivity of a cellular structure composed of a polymer skeleton with conductivity $\kappa_{m}$ impregnated by a fluid (air) of conductivity $\kappa_{f}$. The emphasis is put on the way heat flows across the thickness of a slab with open cells, not in the lateral conductivity tensor of the structure [21,22]. Radiative and convective heat transfers though channels are ignored at this stage. In order to clarify expectations, the thermal conductivity of the PLA nanocomposites filled with small amounts of graphene nanoplatelets or multi-wall carbon nanotubes or both is $\kappa_{m}$ = 0.5–0.6 W/mK [11,14]. A much higher conductivity, up to 3 W/mK, has been achieved in 3D-printed parts produced with LLDPE containing a high concentration of graphene nanoplatelets [23]. These values of $\kappa_{m}$ must be compared to the one of air, $\kappa_{f}$ = 0.026 W/mK in normal conditions. It follows that one may reasonably set $\kappa_{f}=0$ for first-guess evaluation, although calculations presented below have also been performed with a non-zero conductivity of air. As for the geometry, it is assumed that the structure of interest is a slab of thickness *d* periodic in two dimensions, the conductivity being measured along the perpendicular direction *z*. In the (x,y) plane, the unit cell presents an area *S*. These parameters are clarified on a particular case in Figure 1. The holey geometry illustrated there contains bars and holes between bars. The lateral dimensions of the bars and holes are around 1 mm in typical applications.

The objective of this paper is to explore different structures and compare their thermal conductivity efficiency for the same volume-filling fraction, namely the same quantity of nanocomposite per unit volume. In addition, the structures must be suitable for microwave shielding applications. The paper contains two main sections, one devoted to heat conductivity and the other devoted to electromagnetic interference shielding effectiveness.

## 2. Heat Conductivity of Holey Structures

### 2.1. Calculation of the Thermal Conductivity

A finite-difference code has been developed and used to calculate the effective thermal conductivity of a slab with the geometry described in Section 1. The software consists of solving iteratively Laplace’s equation $\nabla^{2}T=0$ for the temperature, starting with an approximate initial distribution. It is assumed that the lower ($z=z_{1}$) and upper ($z=z_{2}$) faces of the slab are in contact with thermostats at temperature $T_{1}$ and $T_{2}$. For the structures of interest here, the local thermal conductivity κ(x,y,z) is a piece-wise constant function of the coordinates taking the values $\kappa_{m}$ inside the skeleton and $\kappa_{f}$ outside it. Suitable boundary conditions are applied on each internal surface of the skeleton, namely *T* is a continuous function of the coordinates, as are the parallel components of $\vec{\nabla }T$ and the normal component of the heat current density. When $\kappa_{f}$ is set to 0, it means that the internal surfaces are insulating. From the solution of Laplace’s equation, the heat power *Q* flowing across the structure in the direction of increasing *z* is calculated and the effective conductivity of the structure follows from the relation

(1) $$\kappa_{e}=-\;\frac{Q(z_{2}-z_{1})}{S(T_{2}-T_{1})}.$$

Approximate expressions of $\kappa_{z}$ are interesting for an exploratory purpose. The Appendix A present a simple homogenization procedure based on 2D averaging (2DA) that appears to be useful. The implicit assumption behind this approach is that the local conductivity κ(x,y,z) for a given *z* value varies much rapidly with *x* and *y* than the temperature derivative ∂T/∂z does. As a consequence, κ(x,y,z) can be homogenized and replaced by its average value over the cross section of the unit cell at coordinate *z*. The result is (see Equation (A4))

(2) $$\kappa_{e}^{-1}=\frac{1}{d}\;\int_{z_{1}}^{z_{2}}\frac{dz}{\phi \left(z\right)\kappa_{m}+\left[1-\phi \left(z\right)\right]\kappa_{f}}\;\mathrm{with}\;d=z_{2}-z_{1}$$

where ϕ(z) is the cumulative relative cross-sectional area of the skeleton at coordinates *z* (total area of the skeleton intercepted by the horizontal plane at coordinate *z* divided by the overall area). Equation (2) is similar to Equation (2) of ref. [25] derived in a different context. Two important bulk properties are related to ϕ(z): the volume-filling fraction $f_{v}$ (fraction of the volume occupied by the skeleton) and its complementary value, the porosity η

(3) $$f_{v}=1-\eta =\frac{1}{d}\;\int_{z_{1}}^{z_{2}}\phi \left(z\right)\;dz=\bar{\phi }.$$

As demonstrated at the end of Appendix A, Equation (2) overestimates the real conductivity of the structure. It is exact when the local conductivity does not depend on the horizontal coordinates *x* and *y* (planar graded structure). In the opposite case where the local κ(x,y,z) does not depend on the coordinate *z*, the structure is composed of walls parallel to the *z* direction. As a result, ϕ(z) is a constant equal to the volume-filling fraction $f_{v}$. From the thermal point of view, the structure can be viewed as an association of two conductances in parallel, one with value $\kappa_{m}f_{v}S/d$ associated with the walls and the other with value $\kappa_{f}\left(1-f_{v}\right)S/d$ associated with the fluid phase. One obtains in that case

(4) $$\kappa_{e}=f_{v}\kappa_{m}+\left(1-f_{v}\right)\kappa_{f}.$$

Equation (2) for a constant ϕ(z) reproduces this expression. Equation (2) is therefore also exact in that particular case. When ϕ(z) varies in a narrow interval around its mean value $f_{v}$, writing $\phi \left(z\right)=f_{v}+\delta \phi \left(z\right)$ and developing the integrand of Equation (2) in power series of δϕ(z) up to the second order yields

(5) $$\kappa_{e}\approx f_{v}\kappa_{m}+\left(1-f_{v}\right)\kappa_{f}-\frac{{\left(\kappa_{m}-\kappa_{f}\right)}^{2}}{f_{v}\kappa_{m}+\left(1-f_{v}\right)\kappa_{f}}\;\bar{\delta \phi^{2}}$$

where $\bar{\delta \phi^{2}}=\left(1/d\right)\int_{z_{1}}^{z_{2}}{\left[\phi \left(z\right)-f_{v}\right]}^{2}\;dz$. The above equation is asymptotically correct at the limit of vanishingly small $\bar{\delta \phi^{2}}$.

### 2.2. Mesh Structures

Mesh structures are composed of interconnected prismatic bars [26], sometimes attached to cubic or spherical nodes. For electromagnetic shielding applications, they often consist of a slice containing one or several layers of a structure periodic in three dimensions, see Figure 1. In the present paper, results for the effective thermal conductivity are given for a single layer, except otherwise stated.

**Figure 2:**
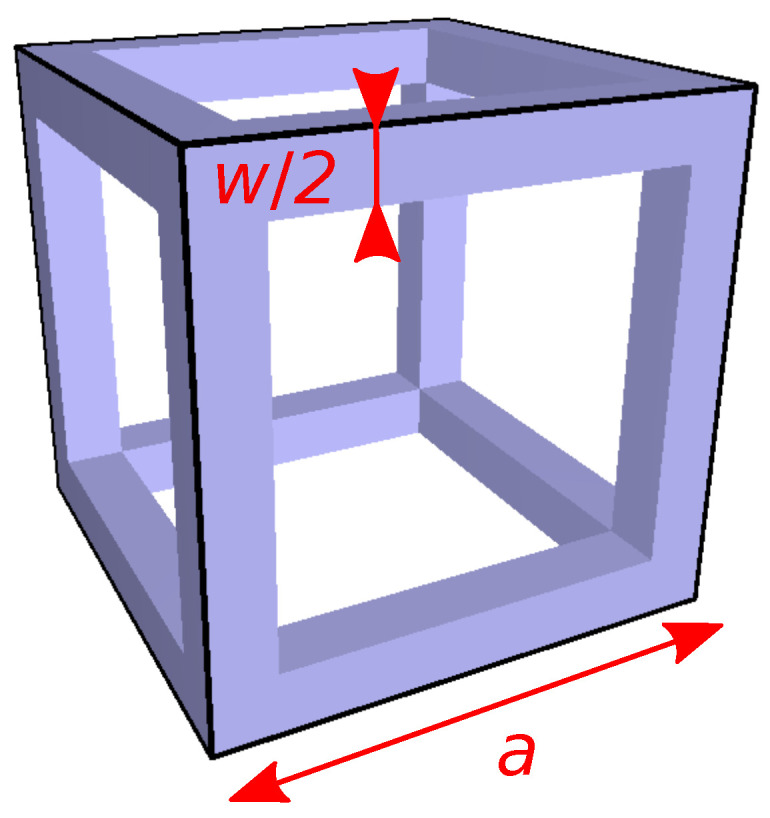
Unit cell of a simple cubic lattice composed of wire cubes assembled side by side (Dul’nev model). In the 3D-lattice, the width of the cube edges is *w*.

Equation (2) is readily applied to the conductivity along [001] of the so-called Dul’nev model [27], which consists of square-based prisms assembled on a simple cubic lattice as illustrated in Figure 2. The result writes [28]

(6) $$\kappa_{e}^{-1}=\frac{1-t}{t^{2}\kappa_{m}+\left(1-t^{2}\right)\kappa_{f}}\;+\;\frac{t}{\left(2t-t^{2}\right)\kappa_{m}+{\left(1-t\right)}^{2}\kappa_{f}}$$

where t=w/a is the ratio between the square edge *w* and the lattice parameter *a*. The parameter *t* is related to the porosity η (see Equation (3)) of the structure. It is a root of the cubic equation [27] $2t^{3}-3t^{2}+1-\eta =0$ that, remarkably enough, can be solved analytically in the form $t=1/2+\cos\left([\arccos(2\eta -1)+4\pi ]/3\right)$.

The effective conductivity increases monotonously from $\kappa_{f}$ to $\kappa_{m}$ when *w* varies between 0 and *a*. There is nothing that can be optimized for heat conduction except to have the largest possible effective conductivity for a given volume-filling fraction $f_{v}$. To assess that, one may introduce a figure of merit (thermal conductivity efficiency) of the structure by the dimensionless ratio [29]

(7) $$\chi =\frac{\kappa_{e}-\kappa_{f}}{f_{v}\left(\kappa_{m}-\kappa_{f}\right)}.$$

To catch the content of this relation, one may refer to the fundamental inequalities [30,31]

(8) $$\frac{1}{\frac{f_{v}}{\kappa_{m}}\;+\;\frac{1-f_{v}}{\kappa_{f}}}\;\leq \;\kappa_{e}\;\leq \;f_{v}\kappa_{m}+\left(1-f_{v}\right)\kappa_{f}.$$

Rearranging the terms involved in the upper limit yields χ≤1. The closest χ to 1, the more efficient the heat transfer is. The lower bound of Equation (8) tells us that

(9) $$\chi \geq \frac{\kappa_{f}}{f_{v}\kappa_{f}+\left(1-f_{v}\right)\kappa_{m}}.$$

**Figure 3:**
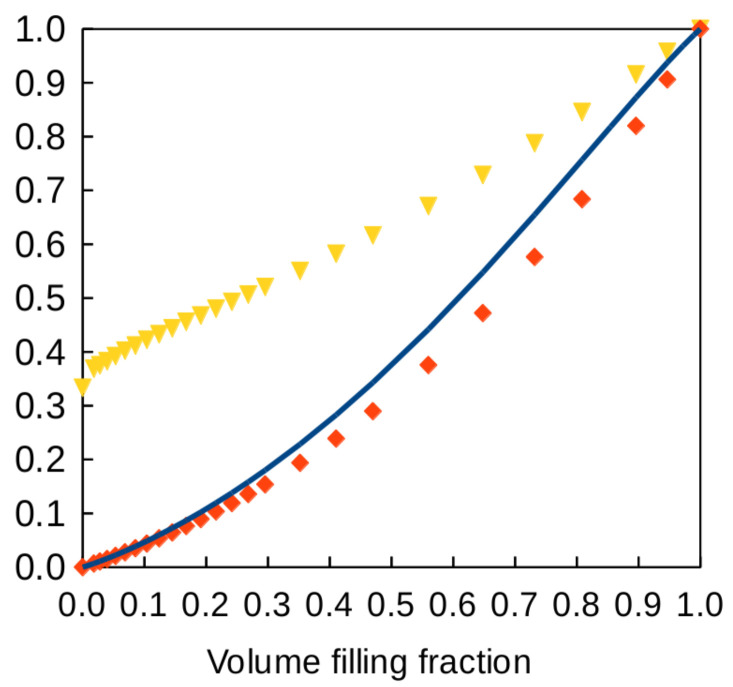
Thermal properties of the Dul’nev lattice versus filling fraction when the conductivity of air is neglected. Blue curve: relative conductivity $\kappa_{e}/\kappa_{m}$ predicted by Equation (6); red diamond: results of full numerical calculations; yellow triangles: thermal conductivity efficiency χ from the full numerical calculations.

Figure 3 displays several results for the Dul’nev lattice in the particular the case where $\kappa_{f}=0$. The blue solid-line curve represents the variation of the ratio $\kappa_{e}/\kappa_{m}$ given by Equation (6) versus volume-filling fraction. The red diamonds display full numerical calculations of $\kappa_{e}/\kappa_{m}$. The yellow triangles are the corresponding numerical values of the thermal conductivity efficiency χ. For small *t*, the blue curve fits the numerical data very well (grid mesh was 100×100×101). The asymptotic behavior of Equation (6) for small *t* is $\kappa /\kappa_{m}=t^{2}+t^{3}+t^{4}/2+O\left(t^{5}\right)$ still under the simplifying assumption $\kappa_{f}=0$. Solving $f_{v}=3t^{2}-2t^{3}$ by perturbation, one obtains $t=\sqrt{f_{v}/3}+f_{v}/9+\left(5/18\right){\left(f_{v}/3\right)}^{3/2}+\cdots$ and, hence, $\chi =\left(1/3\right)[1+\left(5/3\right)\sqrt{f_{v}/3}+13f_{v}/18+\cdots ]$. At the limit of vanishing filling fraction, the efficiency of the lattice is 1/3. This result can be interpreted by the fact that one bar over three in each unit cell conducts heat across the thickness of the structure, the remaining 2/3 do not.

The expression $\kappa_{e}/\kappa_{m}∼{\left(w/a\right)}^{2}$ obtained for small *w* when $\kappa_{f}$ = 0 is asymptotically exact. Indeed, when the lattice is made of bars that are very thin compared to their length, it may be viewed as a resistor lattice. For a simple cubic lattice of thermal resistors of resistance *R*, the effective conductivity is 1/Ra [32]. Substituting $\left(1/\kappa_{m}\right)a/w^{2}$ for *R* reproduces the asymptotic result $\kappa_{e}/\kappa_{m}={\left(w/a\right)}^{2}=f_{v}/3$.

**Figure 4:**
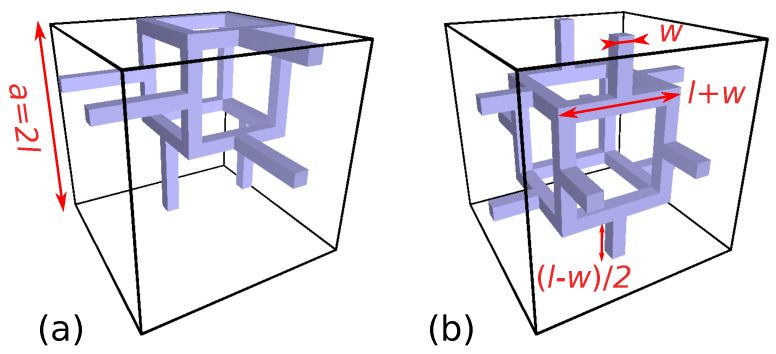
Two representations of the unit cell of the Gibson–Ashby structure. In (**a**), the wire cube is located at a corner of the cubic cell, in (**b**) it is centered in the cell. When heat source and drain are contacted to the top and bottom faces of the unit cell, the layer in (**b**) has a symmetry plane at half thickness whereas the layer (**a**) does not. The effective conductivity values of the two structures differ. Another way to have a structure with a symmetry plane at half layer is to move the structure (**b**) by l=a/2 in the vertical direction. The plane at half thickness will then bisects the vertical stunts. The effective conductivity will be the same as for structure (**b**).

One may expect that any isotropic 3D model should have an efficiency around 1/3 at small volume-filling fraction, in agreement with experimental data for foams [33]. A non-trivial example is the Gibson–Ashby isotropic structure composed of wire cubes interconnected by six stunts, two in each direction (see Figure 4). There are 18 elements in the unit cell displayed in Figure 4a, all assumed to have a square cross section. The volume-filling fraction is $f_{v}=\left[12\left(l+w\right)w^{2}-16w^{3}+6\left(l-w\right)w^{2}\right]/a^{3}$ where *w* is the width of the elements, l=a/2 is half the lattice parameter. The first term in the numerator comes from the 12 elements that form the cube, the second avoids the triple counting of the intersection volumes of the elements at the cube vertices, the third term accounts for the connecting stunts. The result is $f_{v}=\left(9w^{2}l-5w^{3}\right)/4l^{2}$. When $w\ll l,f_{v}∼9w^{2}/4l^{2}$.

Equation (2) applied to this model when $\kappa_{f}=0$ gives

(10) $$\kappa_{e}=\frac{2t^{2}\left(1+t\right)\left(5-t\right)}{15-8t-t^{2}+10t^{3}}$$

with t=w/l. For small *t*, $\kappa_{e}/\kappa_{m}=\left(2t^{2}/3\right)\left(1+4t/3+\cdots \right)=\left(8/27\right)f_{v}\left[1+\left(34/27\right)\sqrt{f_{v}}+\cdots \right]$. The asymptotic value of the thermal conductivity efficiency is therefore χ∼8/27=0.296. It can be noted here that the conductivity of the Dul’nev and Gibson–Ashby lattices for $f_{v}<0.25$ is found to agree reasonably well with the empirical law $\kappa_{e}/\kappa_{m}=f_{v}^{3/2}$ proposed by Gibson and Ashby [34] for low-density foams when the conductivity of air can be neglected. The asymptotic law obtained for these two lattices is $\kappa_{e}/\kappa_{m}∼\alpha f_{v}+\beta f_{v}^{3/2}$, where α and β are two constants close to 1/3 both.

**Figure 5:**
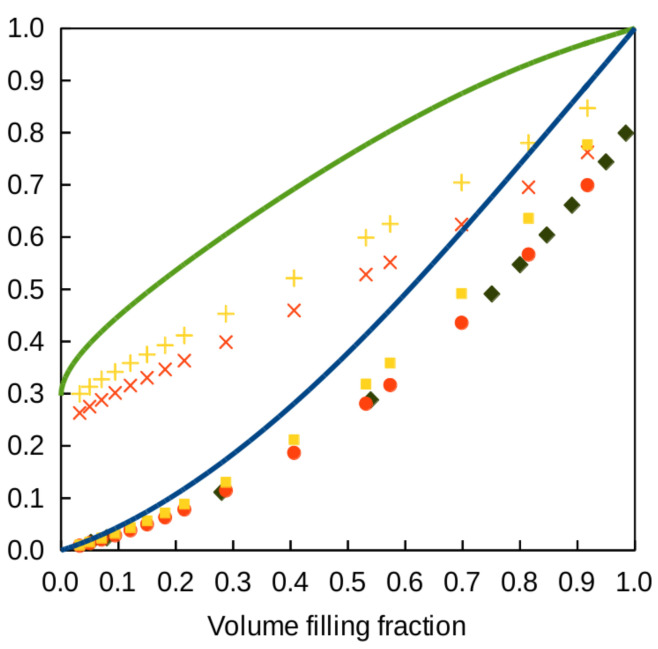
Thermal properties of the Gibson–Ashby lattice model versus filling fraction when the conductivity of air is neglected. Blue curves and green curves: relative conductivity $\kappa_{e}/\kappa_{m}$ and thermal conductivity efficiency χ predicted by Equation (10), respectively; yellow squares and red circles: results of full numerical calculations for the layer of Figure 4a,b, respectively; yellow and red crosses: corresponding thermal conductivity efficiencies χ; blue diamonds: relative conductivity obtained numerically by other authors (see text).

The predictions of the 2DA approximation are compared to numerical calculations in Figure 5. The full-line blue curve represents $\kappa_{e}/\kappa_{m}$ *versus* filling fraction and the full-line green curve is the related thermal conductivity efficiency. The yellow squares and the red circles are the results of full numerical calculations performed for the structure (a) and (b), respectively. The yellow + and the red crosses are the corresponding values of the thermal conductivity efficiency, all well below the prediction of the 2DA approximation. The symmetric layer has smaller effective conductivity (red symbols) than the asymmetric one (yellow symbols). The blue diamonds are numerical results of the relative conductivity found in the literature for the very same symmetric layer [35]. They agree nicely with our own data.

The 2DA approximation can be tricked by some structures such as the woodpile displayed in Figure 1. This structure is made of equidistant prismatic bars, alternatively parallel to the *x* and to the *y* directions. The trick is that ϕ(z) is a constant all across the thickness of the slab under the assumption of identical bars (horizontal thickness *w*, vertical height *h*) arranged on a 2D square lattice (parameter *a*). Equation (2) then leads to Equation (4) with $f_{v}=w/a$, the same as for a heat sink composed of parallel plates of thickness *w* and inter-distance *a*. Of course, crossed horizontal bars, especially at 90°angle, are less efficient to conduct heat in the vertical direction than plain vertical plates would be. This has been assessed by full numerical calculations performed with $\kappa_{f}$ = 0 for three volume-filling fractions, $f_{v}$ = 0.15, 0.25, and 0.35. In each case, three values of the height of the bars were considered, each time with one, two (like in Figure 1), and three layers. The results obtained for $\kappa_{e}/\kappa_{m}$ are collected in Table 1 and must be compared with the value $\kappa_{e}/\kappa_{m}=f_{v}$.

**Figure A1:**
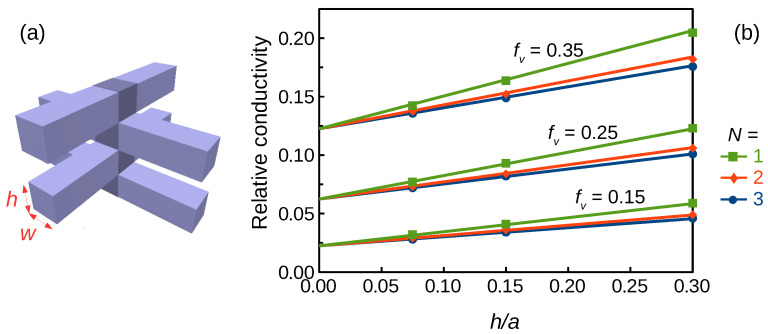
(**a**) Unit cell of the woodpile structure composed of *N* = 2 layers (w/a = 0.15, h/a = 0.15). The part in deep color represents the core column. (**b**) Plot of $\kappa_{e}/\kappa_{m}$ *versus* the height to length ratio h/a of the bars for three volume-filling fractions $f_{v}=w/a$ and for *N* = 1, 2, and 3 layers. The symbols are from Table 1 and the full lines are from Equation (A5).

The numerical values of $\kappa_{e}/\kappa_{m}$ listed in Table 1 are found to be slightly larger than $f_{v}^{2}$, especially for the smallest value of *h*. As described in Appendix B, a woodpile with thin bars behave similarly to a structure composed of one vertical column of section w×w per unit cell (see Figure A1a in Appendix B), which has a relative thermal conductivity $\kappa_{e}/\kappa_{m}={\left(w/a\right)}^{2}$. First-order correction to this value is proportional to $wh/a^{2}$ as demonstrated in Appendix B. This implies that the thermal conductive efficiency of the woodpile structure for vanishingly small filling fraction behaves like χ∼h/a.

### 2.3. Perforated Plates

Perforated plates are widely used structures for heat transfer both by conduction and convection [36]. When the holes keep a constant geometry across the thickness of the plate, Equation (4) for the effective conductivity is exact. The thermal conductive efficiency χ of these structures is one, irrespective of the shape of the holes. When the transverse dimensions of the holes vary slightly across thickness, Equation (5) provides a reliable estimate of $\kappa_{e}$.

### 2.4. Closed-Brick Structure

It follows from the above discussion that the best structure must have most of its skeleton oriented along the *z* axis. With this idea in mind, one is led to open-brick structures containing vertical walls and no horizontal parts. For an open-brick structure with constant walls, Equation (4) is exact. By construction, then, it has a thermal conductivity efficiency χ=1. No other structure can perform better.

**Figure 6:**
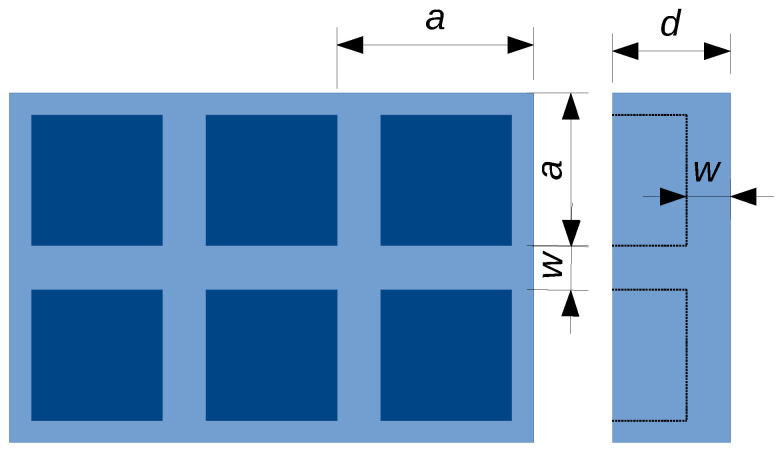
Top view and side view of a closed-brick structure with square lattice.

It is preferable from an electromagnetic point of view, and also to improve the thermal contact with a heat source, to close the structure at one end and to keep it open at the other end to benefit from convection. A simple example is provided by a closed-brick structure. The unit cell of such a structure is depicted in Figure 6 for the case of a square lattice with parameter *a*. The bottom end is closed. For simplicity, it will be assumed that the closing layer and the walls have the same thickness *w*. The 2DA approximation predicts an effective conductivity given by

(11) $$\kappa_{e}^{-1}=\frac{1-f_{z}}{\kappa_{m}-{\left(1-t\right)}^{2}\left(\kappa_{m}-\kappa_{f}\right)}\;+\;\frac{f_{z}}{\kappa_{m}}$$

where $f_{z}=w/d$ and t=w/a.

Calculation have been performed for a constant overall height *d* = 7 mm, the parameter *w* has also been kept constant at 1.25 mm while varying the lattice parameter *a* in a given interval. Air is taken into account in the calculations, assuming $\kappa_{m}/\kappa_{f}=15$. Table 2 lists the result obtained. The structures with *a* smaller than 10 mm demonstrate a good heat transfer effectiveness (χ>0.7). The 2DA approximation works fairly well (accuracy on χ better than 7% in the domain of parameters recorded in Table 2).

### 2.5. Inverse-Pyramid Structure

**Figure 7:**
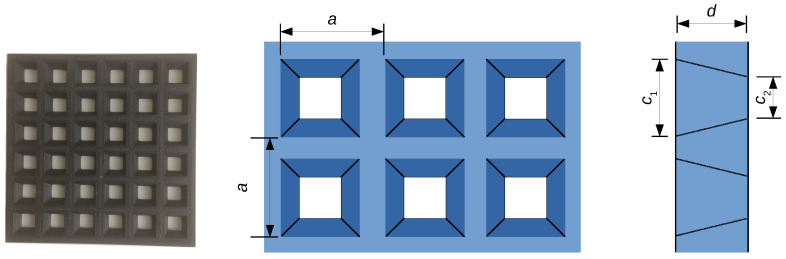
Top view and side view of an open structure with truncated square pyramidal holes. The picture on the left is a 3D-printed model produced with a carbon black filled PLA filament.

From the electromagnetic point of view, holes with constant section that cross the structure may behave like a waveguide. Depending on wavelength, some radiations can therefore be transmitted by the holes. To reduce that transmission, one may imagine holes whose cross section decreases when proceeding down into the structure. A simple example is the so-called inverse-pyramid model illustrated in Figure 7. For simplicity, the 2D unit cell is chosen to be a square of size *a*, the holes are truncated pyramids with square basis, with a lateral size equal to $c_{1}$ and $c_{2}<c_{1}$ on both faces of the slab.

The 2DA approximation is easily applied to this type of structure. Assuming $\kappa_{m}>\kappa_{f}$, Equation (2) leads to

(12) $$\frac{\kappa_{e}}{\kappa_{m}}=\frac{\gamma_{1}-\gamma_{2}}{\mathrm{artanh}\gamma_{1}-\mathrm{artanh}\gamma_{2}}\;\mathrm{with}\;\gamma_{i}=\sqrt{\frac{\kappa_{m}-\kappa_{f}}{\kappa_{m}}}\;\frac{c_{i}}{a}\;,\;i=1,2$$

while the volume-filling fraction is $f_{v}=1-\left(c_{1}^{2}+c_{2}^{2}+c_{1}c_{2}\right)/3a^{2}$. When $c_{1}$ is close to $c_{2}$, Equation (5) yields

(13) $$\frac{\kappa_{e}}{\kappa_{m}}\approx 1-\gamma^{2}-\frac{\gamma^{2}}{1-\gamma^{2}}\;{\left(\gamma_{2}-\gamma_{1}\right)}^{2}\;\mathrm{with}\;\gamma^{2}=\frac{\kappa_{m}-\kappa_{f}}{\kappa_{m}}\;\left(1-f_{v}\right).$$

when $c_{1}=c_{2}$, the thermal conductivity efficiency χ of the structure is readily found equal to 1, as it should. When $c_{1}\approx c_{2}$, $\chi =1-O({\left(\gamma_{1}-\gamma_{2}\right)}^{2})$.

**Figure 8:**
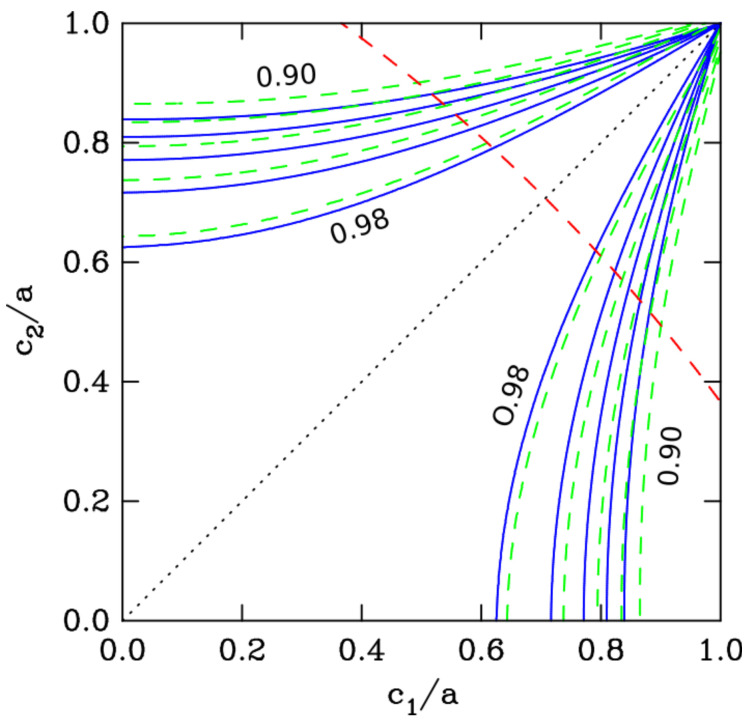
Contour plot of the thermal conductivity efficiency χ of the structure shown in Figure 7 *versus* the geometrical parameters $c_{1}/a$ and $c_{2}/a$. The solid-line blue curves correspond to $\kappa_{f}=0$, the dashed-line green curves correspond to $\kappa_{f}=\kappa_{m}/10$. The dashed-line red curve is the range of parameters for which the volume-filling fraction $f_{v}$ is 0.5. From this curve up to the upper right corner of the figure, $f_{v}>0.5$.

Contour curves of the thermal conductivity efficiency χ of the inverse-pyramid structure are plotted in Figure 8 versus the geometrical parameters $c_{1}/a$ and $c_{2}/a$. Two cases are considered: $\kappa_{f}=0$ (blue solid-line curves) and $\kappa_{f}=\kappa_{m}/10$ (green dashed-line curves). In these two cases, the effect $\kappa_{f}$ has on the contour plot is moderate. The thermal conductivity efficiency remains larger than 0.9 in a great part of the parameter space. The diagonal shown by the dotted line in Figure 8 is the crest curve along which χ = 1.

Full numerical calculations have been performed for an inverse-pyramid model under the assumption $\kappa_{f}$ = 0. The geometrical parameters were *a* = 1.27 cm, *d* = 0.65 cm, $c_{1}/a$ = 0.76 and $c_{2}/a$ = 0.46 (grid mesh was 100×100×61). The filling fraction is $f_{v}$ = 0.6204. The numerical results are $\kappa /\kappa_{m}$ = 0.585 and χ = 0.948 (the 2DA approximation gives 0.6013 and 0.9692, respectively). The very same structure with non-zero air conductivity, $\kappa_{f}=\kappa_{m}/10$, gives $\kappa /\kappa_{m}$= 0.632 and χ = 0.925.

### 2.6. Pin Convective Heat Sink

**Figure 9:**
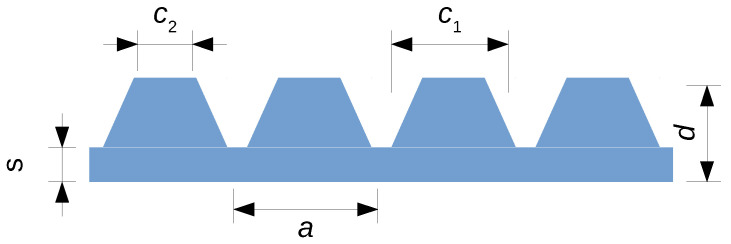
A simple model of pin convective heat skin. The structure is periodic in the two horizontal directions, with lattice parameter a. The protrusions are truncated pyramids with square bases of edge size $c_{1}>c_{2}>0$.

A convective heat sink is often realized by having a set of parallel metallic plates standing perpendicular to a flat basis. Air can flow between the fins, especially when its circulation is forced by a fan. A straight-fin heat sink may not be the best structure for electromagnetic shielding purpose, due to the horizontal anisotropy caused by the fin plates. It would be better to have an array of pins, for instance truncated pyramids mounted on a flat plate, here after called substrate, see Figure 9.

One may repeat the same calculations as for the inverse-pyramid structure with the 2DA approximation. It suffices to exchange the role of $\kappa_{m}$ and $\kappa_{f}$, in Equation (12) with the consequence that the square root in the definition of the parameters $\gamma_{i}$ becomes purely imaginary. As a result, the inverse hyperbolic artanh function transforms in the trigonometric arctan function. Adding the conductivity of the substrate, one is therefore led to

(14) $$\begin{array}{ccc} & & \kappa_{e}^{-1}=\frac{1}{d}\left[\frac{s}{\kappa_{m}}\;+\;\frac{d-s}{\kappa_{f}}\;\frac{\arctan\gamma_{1}-\arctan\gamma_{2}}{\gamma_{1}-\gamma_{2}}\right]\\ & & \;\mathrm{with}\;\gamma_{i}=\sqrt{\frac{\kappa_{m}-\kappa_{f}}{\kappa_{f}}}\;\frac{c_{i}}{a}\;i\;=1,2. \end{array}$$

when $c_{2}/a\gg \sqrt{\kappa_{f}/\left(\kappa_{m}-\kappa_{f}\right)}$, which may happen when $\kappa_{f}$ is negligible compared to $\kappa_{m}$, Equation (14) becomes

(15) $$\kappa_{e}^{-1}\approx \frac{1}{d}\left[\frac{s}{\kappa_{m}}\;+\;\frac{d-s}{\kappa_{m}-\kappa_{f}}\;\frac{a^{2}}{c_{1}c_{2}}\;\left[1-{\left(\frac{\kappa_{f}}{\kappa_{m}-\kappa_{f}}\;\frac{a^{2}}{c_{1}c_{2}}\right)}^{2}\;\frac{c_{1}^{2}+c_{1}c_{2}+c_{2}^{2}}{3a^{2}}\right]\right].$$

Full numerical calculations have been performed for a structure with *a* = 6 mm, *s* = 1.5 mm, *d* = 5.7 mm, pyramid height = 4.2 mm, $c_{1}$ = 4.8 mm and $c_{2}$ = 0.6 mm (100 × 100 × 97 mesh). The volume-filling fraction is $f_{v}$ = 0.442. When the thermal conductivity of air is 1/15 that of the skeleton ($\kappa_{m}/\kappa_{f}$ = 15), the effective conductivity of the structure is $0.215\kappa_{m}$, with a conductive efficiency χ=0.359 (Equation (14) predicts $\kappa_{e}/\kappa_{m}$ = 0.249 and χ = 0.441). Less obtuse pyramids have also been considered by taking $c_{1}$ = 2.4 mm and $c_{2}$ = 0.72 mm, which reduces the filling fraction to 0.319. Numerical calculations have led to $\kappa_{e}/\kappa_{m}$ = 0.151 and χ = 0.284 (to be compared with 0.162 and 0.322, respectively, with Equation (14)). For both structures, the predictions of the 2DA approximation are fair. The conductivity efficiency of both structures obtained numerically is around 0.30, which is not bad, although the effective conductivity is only between 15% and 21% of what could be obtained with a plain sample of the same thickness. Of course, the heat transport could be much better by forced convection.

### 2.7. Foam-Like Structure

There exist several classical models of 3D foam-like structures, such as those based on triply periodic minimal surfaces (TPMS). A well-known TPMS is the so-called P-Schwarz lattice (“P” for primitive), which has demonstrated better performance than the D Schwarz (“D” for diamond) lattice and the gyroid lattice [37,38,39]. Based on these observations, only the first model has been considered in the present paper. The P-Schwarz lattice is a three-periodic structure based on a minimal surface (surface with constant mean curvature) with simple cubic translation symmetry. The structure can be defined by the expression

**Figure 10:**
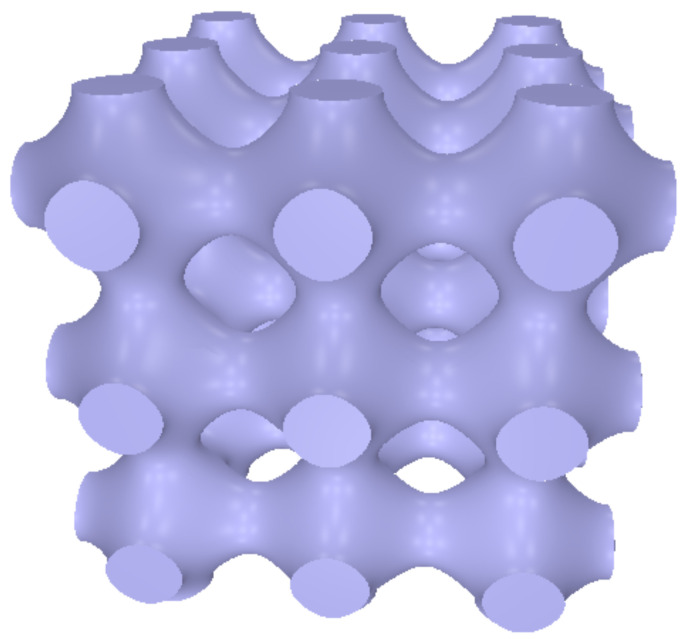
Three-dimensional rendering of the P-Schwarz lattice for *t* = 0.1 (Equation (16)). Three periods are represented along the three axes.

(16) cos(2πx/a)+cos(2πy/a)+cos(2πz/a)>t

where *a* is the lattice parameter and *t* is a parameter related to the volume-filling fraction. The filling fraction is 1 for t≤−3 and 0 for t≥3. It varies continuously in between. Figure 10 illustrates the structure obtained for *t* = 0.1.

The cumulative cross-sectional area of the skeleton at coordinate *z*, ϕ(z), cannot be derived analytically for this structure. However, the results of numerical calculations can be reproduced accurately with the rational expressions

$$\begin{array}{ccc} \phi (z) & = & 1\;\mathrm{for}\;g\leq -1 \end{array}$$

(17) $$\begin{array}{ccc} \phi (z) & = & 1\;-\;\frac{1+g}{\pi }\left[1+\left(\frac{\pi }{2}-1\right){\left(1+g\right)}^{2}\right]\;\mathrm{for}\;-1\leq g\leq 0 \end{array}$$

(18) $$\begin{array}{ccc} \phi (z) & = & \frac{1-g}{\pi }\left[1+\left(\frac{\pi }{2}-1\right){\left(1-g\right)}^{2}\right]\;\mathrm{for}\;0\leq g\leq 1 \end{array}$$

$$\begin{array}{ccc} \phi (z) & = & 0\;\mathrm{for}\;g\geq 1 \end{array}$$

where g=[t−cos(2πz/a)]/2. If there is a coordinate *z* such that g>1, the solid phase is broken in isolated islands and no longer forms a percolating skeleton. Avoiding this unrealistic situation requires t<1. Similarly, the structure is permeable when t>−1.

Integrating ϕ(z) with respect to *z* over one period *a* of the structure yields the volume-filling fraction $f_{v}$. In the interval −1<t<1, $f_{v}$ was found to vary quasi linearly with *t*, the relation obtained numerically being $f_{v}=0.5-0.2841\;t$, in excellent agreement with results obtained by Ronca et al. [37].

**Figure 11:**
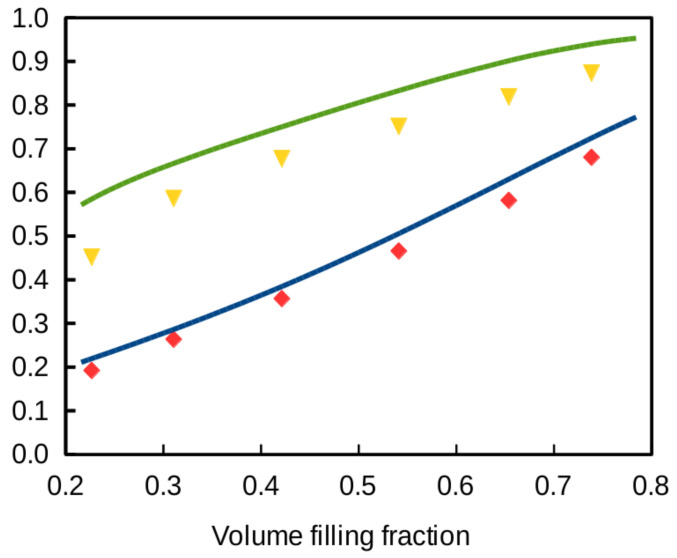
Thermal properties of the P-Schwarz lattice versus filling fraction when the conductivity of air is one tenth of that of the skeleton. Blue and green curves: relative conductivity $\kappa_{e}/\kappa_{m}$ predicted by Equation (2) and the corresponding thermal conductivity efficiency χ (Equation (7)); red diamonds: $\kappa_{e}/\kappa_{m}$ from full numerical calculations; yellow triangles: χ from full numerical calculations.

With these elements in hands, Equation (2) is easily applied to characterize the thermal properties of the structure. The full-line curves in Figure 11 show the effective conductivity and the thermal conductivity efficiency versus filling fraction when air conductivity is set to $\kappa_{m}/10$. The markers are the result of full numerical calculation performed on a 100 × 100 × 101 mesh. The 2DA approximation slightly overestimates the conductivity. The P-Schwarz structure behaves similarly as other simple cubic lattices investigated above. Its thermal conductivity efficiency is above 70% whenever $f_{v}$ exceeds 0.5. Interestingly, the thermal conductivity efficiency χ is still around 0.45 at the limit where the skeleton splits into disconnected islands ($f_{v}$ = 0.2159). Of course, this result is due to air conductivity, otherwise χ would drop to zero at the non-percolation limit.

On the experimental side, the effective conductivity of the P-Schwarz and other similar structures produced by SLS have been investigated for different values of the porosity. The 3D-printing feeding material was a powder of graphene-wrapped thermoplastic polyurethane (TPU). In particular, $\kappa_{e}/\kappa_{m}$ = 0.37 has been measured for the P-Schwarz model with 60% porosity [37], which agrees well with the calculations plotted in Figure 11 for $f_{v}$ = 0.4.

## 3. Microwave Shielding Properties

The electromagnetic properties of the structure illustrated in the photography of Figure 7 have been measured in the Ka band (26–37 GHz). The 3D printed model was obtained from a PLA filament containing 25 wt% of carbon black grade N121 produced by “OMSK carbon group”. Carbon black filled PLA filaments were produced by melt mixing at 180 °C using a twin-screw extruder in a two-stage process. Pellets of the black carbon/PLA compound were fabricated first. Then the filaments were produced from the compound after the pellets were dried at 80 °C for 4 h. Filaments with 1.75±0.05 mm nominal diameter were extruded. Printing was performed on Creatbot F430 3D-printer with following parameters: 0.4 mm nozzle diameter; 0.1 mm layer height and 0.35 mm layer width; 10 mm/s printing speed; retraction switched off; printing without raft; 110% extrusion speed; extruder temperature 215 °C; bed temperature 40 °C; chamber temperature 30 °C.

**Figure 12:**
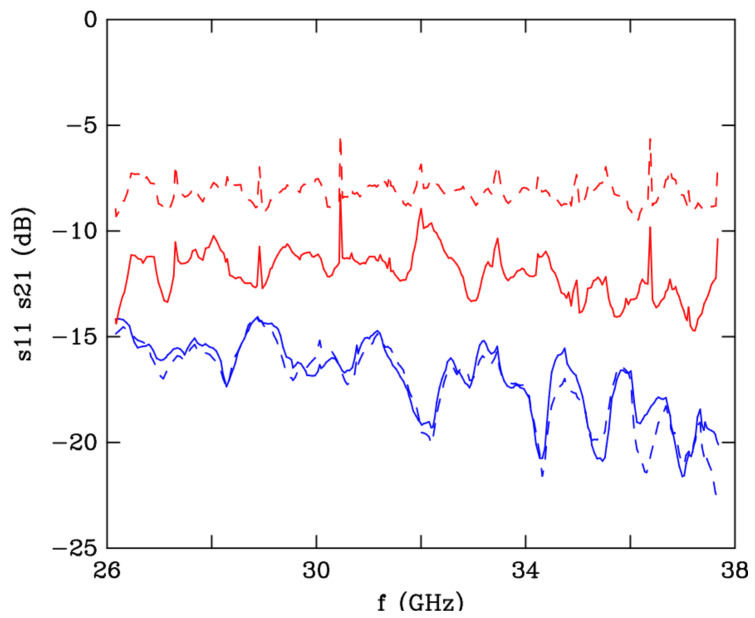
Variation *versus* frequency of the s11 (red curves) and s21 (blue curves) signals in dB of the structure with truncated pyramidal holes shown in the photo of Figure 7. The full-line curves correspond to the waves arriving from the largest basis of the holes, the dashed-line curves are for the reversed direction. The geometrical parameters are: *d*= 6.5 mm, *a* = 13.1 mm, $c_{1}$ = 9.0 mm, and $c_{2}$ = 6.0 mm (see Figure 7).

The electromagnetic measurements were realized with a scalar network analyzer Elmika R2-408R in free-space configuration, using two horn antennas WP-07 (for a detailed description, see ref. [40]). The 3D-printed sample was placed between the horn antennas and the relative amplitudes of the reflected (s11) and transmitted (s21) radiations were measured. The scattering parameters are plotted in Figure 12 for two orientations of the sample: the waves enter the plate from the side where the holes are larger (full-line curves) and from the other side (dashed-line curves). There is a 5 dB attenuation of the reflection signal s11 when the waves travel in the direction of increasing material density (full-line red curve). This is because the contrast of refraction index in comparison to air is more progressive this way than the other way round. The pyramidal hole geometry behaves similarly as a stack of plain layers containing an increasing concentration of nanofillers [8]. By contrast, the transmission signal s21 is independent of the traveling direction, thanks to time-reversal symmetry. It varies between −15 dB and −21 dB across the frequency interval of the Ka band. The average shielding efficiency in transmission around −17 dB. The shielding of the incident radiations is primarily due to dielectric losses and secondary to reflection (red curves in Figure 12). The absorption is due to heat dissipation in carbon black particles whose electromagnetic coupling by capacitive effects is expected to be efficient at microwave frequencies, much more than their resistive coupling at zero frequency. By comparison, the same structure printed with pure PLA has an average s21 parameter around −4 dB.

The electromagnetic properties of structures based on the Gibson–Ashby model (Figure 4b) have been investigated theoretically by finite elements [41]. Meshes containing one, two, and three layers of periodic cells with 3 mm lattice parameter and *w* = 0.83 mm were explored. High broadband absorption (power transmission below 1% ) was predicted in the Ka band for all three structures, already with on a moderately conductive skeleton (1–200 S/m) [9].

Woodpile-like frameworks have been produced by solvent-cast 3D printing with highly conductive (5000 S/m) inks based on a nanotube/PLA nanocomposite. Shielding efficiency better than 20 dB was measured on different structures with mm-sized lattice parameter and sub-mm thickness [42]. Shielding properties of a woodpile structure produced by FDM 3D printing with conducting (0.63 S/m) ABS have been reported by other authors [43]. The structure was a pile of three orthogonal rows of rectangular prisms of 1.2 mm height each. It demonstrated slightly better electromagnetic performance than a perforated slab of the same overall thickness. A power attenuation better than 90% in a large band centered on 6.0 GHz (λ/4 plate) was reported.

Anechoic plates with a pyramid array of the kind displayed in Figure 9 ($a=c_{1}$ = 2.5 mm, $c_{2}$ = 0, *d* = 3.5 mm, *s* = 1 mm) have been made by FDM 3D printing with different composite polymers [44]. The structures were investigated in the C band (3.5–7.0 GHz) of the electromagnetic spectrum. An average absorption of 20 dB all over the C band was achieved through a 3D pyramid array printed with a graphene-doped PLA filament of 86 S/m electrical conductivity.

Shielding effectiveness of novel structures containing a P-Schwarz conducting sheet embedded in a polymer matrix has been investigated numerically by finite-element method [45]. Here, unlike the model illustrated in Figure 10, the P-Schwarz surface cos(2πx/a)+cos(2πy/a)+cos(2πz/a)=t associated with a fixed value of the parameter *t* (see Equation (16)) is thickened. The volume-filling fraction of the conductive material can be changed almost at will by varying the thickness of the sheet. Promising results have been predicted and attributed to the continuous, smooth surface of the conducting network, by opposition to what is realized with joints of conductive struts.

## 4. Conclusions

Both approximate calculations based on a simple homogenization process and full numerical calculations have been performed to compare thermal conductive properties of different holey structures. The effective conductivity of the woodpile framework derived in Appendix B attests to its poor heat conduction efficiency. Mesh cubic lattices and the anechoic pyramidal layer perform better, although they are not the best conductive structures among those examined in this paper. For a filling fraction around 0.5, their thermal conductivity efficiency χ is ∼0.5 for the cubic mesh lattices and ∼0.3 for the pin heat sink, which yields an advantage to the mesh network, more especially the Dul’nev lattice of wires (Figure 2). The P-Schwarz foam lattice is better than the mesh lattices in view of its thermal conductive efficiency (χ≈0.7) at half volume-filling fraction. Better thermal performance is achieved with the closed-brick and the inverse-pyramid models. Still with a filling fraction of 0.5, both models may demonstrate a thermal conductivity efficiency above 0.8. The advantage of the latter structure over the former is its permeability to air.

In this work, the electromagnetic properties of a structure with truncated pyramidal holes fabricated with black carbon-filled PLA polymer has been characterized experimentally. This structure appears to be interesting for both electromagnetic shielding and heat conductivity effectiveness. A short review of results reported in the literature for other holey structures has been presented. Combining these data with those related to heat conductivity demonstrates the multifunctional character of these structures.

## Figures and Tables

**Table 1:** Relative effective conductivity $\kappa_{e}/\kappa_{m}$ obtained by full numerical calculations for woodpile structures composed of *N* = 1, 2, and 3 layers and for three values of $f_{v}$. For each structure, three values of the height *h* of the bars were considered. The conductivity $\kappa_{f}$ of the fluid has been set to zero. The step size of the discretization grid along *z* was kept the same for all calculations; the mesh used for the thicker slab (h/a = 0.3 and *N* = 3) was 100×100×361.

		$f_{v}$ = 0.15	$f_{v}$ = 0.25	$f_{v}$ = 0.35
N	h/a	$\kappa_{e}/\kappa_{m}$		
1	0.075	0.032	0.077	0.142
	0.150	0.041	0.093	0.164
	0.300	0.059	0.123	0.205
2	0.075	0.029	0.073	0.137
	0.150	0.036	0.084	0.152
	0.300	0.049	0.106	0.182
3	0.075	0.028	0.072	0.136
	0.150	0.034	0.082	0.149
	0.300	0.046	0.101	0.176

**Table 2:** Relative conductivity $\kappa_{e}/\kappa_{m}$ and thermal conductivity efficiency χ for a closed-brick structures in air, assuming $\kappa_{m}=15\;\kappa_{f}$. The structure has a square lattice with varying parameter *a*. Its overall height is *d* = 7 mm, all the walls and the closing layer have a thickness of 1.25 mm.

Parameters		Full Numerical Results		2DA Approximation	
a (mm)	$f_{v}$	$\kappa_{e}/\kappa_{m}$	χ	$\kappa_{e}/\kappa_{m}$	χ
4	0.612	0.594	0.923	0.607	0.946
5	0.538	0.509	0.881	0.524	0.911
6	0.485	0.447	0.841	0.463	0.876
7	0.446	0.401	0.804	0.417	0.842
8	0.415	0.365	0.770	0.381	0.810
9	0.391	0.336	0.738	0.351	0.780
10	0.371	0.312	0.709	0.327	0.752
12	0.341	0.276	0.658	0.290	0.701
*∞*	0.179			0.080	0.080

## Appendix A. 2D Homogenization for the Thermal Conductivity

Homogenization is practical approach used to calculate a macroscopic property of a system when a material parameter fluctuates on a scale much smaller than the system dimensions. It will be applied at first order to the heat continuity equation $\vec{\nabla }\cdot \vec{u}=0$ where $\vec{u}=-\kappa \vec{\nabla }T$ is the heat current density. The geometry of the system of interest is a right prism with its bases parallel to the (x,y) plane. They are located at $z=z_{1}$ and $z=z_{2}$. They are assumed to be kept at uniform and constant temperature $T_{1}$ and $T_{2}$, respectively. Either the prism is the unit cell of a 2D-periodic structure (lateral periodic boundary conditions) or its lateral surface is covered by an insulating material ($\vec{u}\cdot \vec{n}=0$ on the lateral surface, $\vec{n}$ being the unit outward normal to it).

Integrating the heat continuity equation over the section of the unit cell intercepted by the horizontal plane at position $z\in (z_{1},z_{2})$ leads to $\int_{C}\left(u_{x}n_{x}+u_{y}n_{y}\right)ds+{\;\int \int \;}_{S}\partial u_{z}/\partial z\;dxdy=0$. Here *S* is the basis surface, *C* is its boundary curve (perimeter), and $ds=\sqrt{dx^{2}+dy^{2}}$. The line integral over *C* vanishes under the assumed boundary conditions on the lateral surface. One is therefore left with

(A1) $${\;\int \int \;}_{S}u_{z}dxdy=-{\;\int \int \;}_{S}\kappa \frac{\partial T}{\partial z}dxdy=Q$$

where *Q* is a constant independent of the considered *z* coordinate.

It will be convenient to rewrite the equations just obtained as ${\langle \kappa \partial T/\partial z\rangle }_{z}=-Q/S$ where the notation ${\langle f\rangle }_{z}=\left(1/S\right){\;\int \int \;}_{S}f\left(x,y,z\right)\;dxdy)$ is used to denote the value of a function *f* averaged over the cross section of the system at coordinate *z*. Here, *f* is the product of κ and ∂T/∂z. If the local κ does not depend on the *x* and *y* coordinates, it can be put in front of the 2D-integral. In the other cases, the following approximation will be used: ${\langle \kappa \partial T/\partial z\rangle }_{z}\approx {\langle \kappa \rangle }_{z}{\langle \partial T/\partial z\rangle }_{z}$. In the framework of homogenization, this approximation may mean that κ fluctuates with *x* and *y* around its average value on a much smaller spatial scale than ∂T/∂z does. Under this approximation, Equation (A1) becomes

(A2) $$\frac{d{\langle T\rangle }_{z}}{dz}=-\;\frac{Q}{S{\langle \kappa \rangle }_{z}}.$$

Straightforward integration yields

(A3) $${\langle T\rangle }_{z}=-\;\frac{Q}{S}\;\int_{z_{1}}^{z}\frac{d\zeta }{{\langle \kappa \rangle }_{\zeta }}+D$$

with *D* an arbitrary constant. Due to the boundary conditions on both bases, ${\langle T\rangle }_{z_{1}}=T_{1}$ and ${\langle T\rangle }_{z_{2}}=T_{2}$. From there on, the two constant *Q* and *D* can be calculated. Identifying *Q* with its definition Equation (1)) leads to

(A4) $$\kappa_{e}^{-1}=\frac{1}{z_{2}-z_{1}}\;\int_{z_{1}}^{z_{2}}\frac{dz}{{\langle \kappa \rangle }_{z}}.$$

This is the main result of this Appendix. For the structure of interest here, κ(x,y,z) for a fixed value of *z* is a piece-wise constant function of *x* and *y* taking the values $\kappa_{m}$ or $\kappa_{f}$. As a result, ${\langle \kappa \rangle }_{z}$ can be written as $\phi \left(z\right)\kappa_{m}+\left[1-\phi \left(z\right)\right]\kappa_{f}$ where ϕ(z) is the overall cross-sectional area of the skeleton at coordinates *z* divided by the area *S* of the unit cell basis. Equation (2) is thereby demonstrated.

The denominator in the integral of Equation (2) stands for the effective conductivity of the infinitesimal slice of the structure lying in the interval (z,z+dz). According to Wiener’s inequalities (Equation (8)), the real conductivity of the slice at coordinate *z* is less than or equal to $\phi \left(z\right)\kappa_{m}+\left[1-\phi \left(z\right)\right]\kappa_{f}$. As a consequence, $\kappa_{e}$ calculated by Equation (2) is an upper bound of the effective conductivity of the structure.

## Appendix B. Thermal Conductivity of the Woodpile Structure

Woodpile is an important structure in the context of FDM 3D-printing, when filaments are deposited in successive layers along orthogonal directions with infill density smaller than 100%. An approximate expression for the relative conductivity of the woodpile structure is developed here. The unit cell is composed of 2N horizontal bars of length *a* placed on top of each other, each bar being orthogonal to the ones below and above it. The cross section of the bars is a rectangle of width *w* and height *h*. The core of unit cell is the plain vertical column with square cross section w×w that intercepts all the bars (see Figure A1). The bars form branches perpendicular to the core column.

The bars at the bottom of the slab have their down face kept at temperature $T_{1}$. Similarly, the bars at the top have their up face kept at temperature $T_{2}$. The heat flows directly across the structure through the columns. Part of the heat also flows along the topmost bars in the direction of the nearest core column. The same arises at the lowest part of the woodpile. The heat current penetrates partly into the other bars.

From the thermal point of view, a column can be viewed as made of 2N sections of height *h*, each with a lateral branch. Each section has a conductance $G_{0}=\kappa_{m}w^{2}/h$. The side branch increases the conductivity of the section to $G_{0}+G_{1}$ for the terminal sections (those in contact with the heat source and heat sink) and to $G_{0}+G_{2}$ for the 2N−2 inner sections. Accordingly, the thermal resistance of the unit cell is $R=2/\left(G_{0}+G_{1}\right)+\left(2N-2\right)/\left(G_{0}+G_{2}\right)$. It is identified with $R=2Nh/\kappa_{e}a^{2}$. The effective thermal conductivity $\kappa_{e}$ follow from there:

$$\kappa_{e}^{-1}=\frac{a^{2}}{N\kappa_{m}w^{2}}\left(\frac{1}{1+\alpha_{1}h}+\frac{N-1}{1+\alpha_{2}h}\right)$$

where $\alpha_{i}=G_{i}/\kappa_{m}w^{2}$, i=1,2. The contributions of the branches, $\alpha_{i}h$, are supposed to be small. Therefore, the calculations can be limited to terms of first order in these small quantities. In these conditions, one obtains $\kappa_{e}/\kappa_{m}=f_{v}^{2}\left[1+\alpha_{1}h/N+\left(N-1\right)\alpha_{2}h/N\right]$, where $f_{v}=w/a$ is the volume-filling fraction. The parameter $\alpha_{i}$ contains a factor $G_{i}$ that is proportional to the thermal conductivity $\kappa_{m}$ of the material and to the width *w* of the bars. Writing $G_{i}=\beta_{i}\kappa_{m}w$, where $\beta_{1}$ and $\beta_{2}$ are two new dimensionless parameters, one arrives at

(A5) $$\frac{\kappa_{e}}{\kappa_{m}}=f_{v}^{2}+\frac{\beta_{1}+\left(N-1\right)\beta_{2}}{N}\frac{h}{a}f_{v}.$$

The parameters $\beta_{1}$ and $\beta_{2}$ have been adjusted to the results of numerical calculations. The data of Table 1 are well reproduced by using $\beta_{1}$ = 0.80 and $\beta_{2}$ = 0.37, see Figure A1.
